# Role of circulating biomarkers in spinal muscular atrophy: insights from a new treatment era

**DOI:** 10.3389/fneur.2023.1226969

**Published:** 2023-11-13

**Authors:** Querin Giorgia, Marta Gomez Garcia de la Banda, Piera Smeriglio

**Affiliations:** ^1^APHP, Service de Neuromyologie, Hôpital Pitié-Salpêtrière, Centre Référent pour les Maladies Neuromusculaires Nord/Est/Ile de France, Paris, France; ^2^Institut de Myologie, I-Motion Clinical Trials Platform, Paris, France; ^3^European Reference Center Network (Euro-NMD ERN), Paris, France; ^4^APHP, Pediatric Neurology Department, Hôpital Armand Trousseau, Centre Référent pour les Maladies Neuromusculaires Nord/Est/Ile de France, Paris, France; ^5^APHP, Pediatric Neurology and ICU Department, Université Paris Saclay, DMU Santé de l'Enfant et de l'Adolescent, Hôpital Raymond Poincaré, Garches, France; ^6^Centre of Research in Myology, Institute of Myology, Sorbonne Université, INSERM, Paris, France

**Keywords:** SMA, circulating biomarkers, individualized medicine, neurofilaments, miRNA, innovative treatments, gene therapy

## Abstract

Spinal muscular atrophy (SMA) is a lower motor neuron disease due to biallelic mutations in the *SMN1* gene on chromosome 5. It is characterized by progressive muscle weakness of limbs, bulbar and respiratory muscles. The disease is usually classified in four different phenotypes (1–4) according to age at symptoms onset and maximal motor milestones achieved. Recently, three disease modifying treatments have received approval from the Food and Drug Administration (FDA) and the European Medicines Agency (EMA), while several other innovative drugs are under study. New therapies have been game changing, improving survival and life quality for SMA patients. However, they have also intensified the need for accurate biomarkers to monitor disease progression and treatment efficacy. While clinical and neurophysiological biomarkers are well established and helpful in describing disease progression, there is a great need to develop more robust and sensitive circulating biomarkers, such as proteins, nucleic acids, and other small molecules. Used alone or in combination with clinical biomarkers, they will play a critical role in enhancing patients’ stratification for clinical trials and access to approved treatments, as well as in tracking response to therapy, paving the way to the development of individualized therapeutic approaches. In this comprehensive review, we describe the foremost circulating biomarkers of current significance, analyzing existing literature on non-treated and treated patients with a special focus on neurofilaments and circulating miRNA, aiming to identify and examine their role in the follow-up of patients treated with innovative treatments, including gene therapy.

## 1. Introduction

5q-Spinal muscular atrophy (SMA) is a lower motor neuron (LMN) disease characterized by anterior horn degeneration in the brainstem and spinal cord (SC), muscle weakness and atrophy. It occurs in both pediatric and adult forms with symptoms manifesting from birth (SMA type 0 and 1) to adulthood (SMA type 4) (1). SMA is caused by recessive deletions or loss-of-function mutations in the SMN1 gene (chromosome 5q11.2-q13.3), with an incidence of 1 in 10,000 live births and a carrier frequency of about 1 in 50 (2–5). Complete dysfunction of *SMN1* is necessary to determine the SMA phenotype (6–8), with gene deletions accounting for 95% of SMA cases. About 3% of the patients are compound heterozygotes for a deletion and an intragenic point mutation (9). The *SMN2* gene, a centromeric paralog of *SMN1*, is the main genetic modifier of the SMA phenotype and disease severity: multiple copies of *SMN2* may be present in the genome (up to 5 or 6 in SMA type 3 and 4), with a higher number inversely correlating with disease severity (10, 11). The *SMN2* gene sequence is almost identical to that of *SMN1*, except for a few nucleotides causing a differential splicing of the gene leading to the production of a truncated and non-functional SMN protein and only to a small amount of residual functional SMN. This event is sufficient to partially compensate for the functional protein loss due to *SMN1* mutations.

The SMN protein is highly conserved and widely expressed both in the cytoplasm and the nucleus of cells in all somatic tissues and particularly abundant in motor neurons (12). The complex functions of the SMN protein span from mRNA splicing and snRNP assembly to mRNA trafficking and translation (13). Therefore, loss of SMN expression has important consequences on various aspects of cellular and tissue functions (14–16).

From a clinical point of view, SMA is characterized by irreversible and progressive muscle weakness of axial, limb and bulbar muscles (17, 18). SMA patients exhibit considerable clinical heterogeneity and are traditionally classified in four groups based on the age at onset and maximal motor milestones achieved (with type 1 being the most severe and early onset form, and type 4 being an adult and milder form) (19, 20).

In recent years, significant efforts have been made in the development of new therapies for 5q-SMA (21), resulting in the approval of three disease-modifying treatments by the FDA and EMA. These innovative therapeutic approaches aim either at increasing SMN protein circulating levels by mediating alternative *SMN2* splicing (antisense oligonucleotides as nusinersen and small molecules as risdiplam) (22, 23) or at replacing the mutated *SMN1* gene (viral vector mediated gene therapy) (24). Other strategies, such as neuroprotective drugs and molecules to enhance muscle strength (e.g., several anti-myostatin drugs) (25, 26) are also under investigation. All these new therapies have transformed the natural history of the disease, leading to improved survival in patients (particularly in type 1) and to the emergence of new phenotypes, with varying neuromuscular prognoses (27, 28). Clinical evolution of treated and non-treated patients is usually performed using neuromuscular evaluations of muscle strength, motor capacities and bulbar and respiratory function. Clinical tools such as SC imaging (29), muscle MRI (30, 31) and neurophysiology (motor unit count) (32, 33) are being explored as possible biomarkers for the follow-up of patients. However, more specific, and sensitive biomarkers capable of assessing the effect of the treatment and of predicting disease trajectories over time are still partially lacking. Moreover, clinical heterogeneity and variability of therapeutic response between treated patients are still a major challenge in SMA. Therefore, there is a growing emphasis on the search for accurate and reliable molecular circulating biomarkers to better classify patients, guide personalized treatment decisions and grant an appropriate post treatment follow-up (34–36).

The objective of this review is to evaluate the latest developments in the rapidly advancing field of molecular biomarkers for SMA, with a particular focus on indicators that have proven useful for both natural history studies and post-treatment investigations. Different putative predictive biomarkers and their role in treated patients are summarized in Table 1.

**Table 1:** Summary of relevant putative biomarkers for untreated and treated SMA patients.

Biomarker	Tissue	Monitoring and prognostic biomarker	Predictive and responsa biomarker
*SMN2* copy number	Intracellular	Fewer copies of *SMN2* gene correlate with less SMN protein and more severe/advanced disease	No modification after therapy
SMN2-FL mRNA	Intracellular (PBMC, lymphoblasts, fibroblasts, …), blood, skeletal muscle	Lower levels in SMA mice compared to controls Variable levels in different body tissues Correlation with disease severity is unclear	Increased blood levels after treatment with risdiplam Increased blood levels after treatment with nusinersen
SMN2-D7 mRNA	Intracellular (PBMC, lymphoblasts, fibroblasts, …), blood	Inconsistent results	Inconsistent results, probably reduced in blood after treatment with valproic acid
SMN protein	Intracellular (PBMC, lymphoblasts, fibroblasts, …), skeletal muscle, CSF, plasma, urine	Lower levels of circulating SMN protein correlate with more severe/ advanced disease SMN protein levels seem stable over disease progression	Increase of protein levels are observed after treatment (Risdiplam, Nusinersen) in different tissues (with high variability within different tissues)
Creatinine	Serum	Inversely associated with disease severity Higher in type 3 than in type 1 patients Higher in patients with chronic SMA	Increased levels after treatment with nusinersen
CPK	Serum	Slightly elevated in all SMA types Higher levels in milder forms of SMA	Decreased levels after treatment with nusinersen
Neurofilaments	CSF, serum, and plasma	Higher levels in type 1 patients compared to controls and other SMA types Only slightly elevated in chronic type 2 and 3 adolescent and adult patients Higher levels correlate with axonal degeneration and disease severity	In children, decrease of CSF, plasma, and serum levels after treatment with nusinersen Temporary increased followed by decreased levels after AAV-mediated gene therapy
MicroRNA	Serum, SC, skeletal muscle	Differential expression of several miRNA according to SMA severity	Modified regulation of different miRNA after treatment with nusinersen
Omics	CSF, serum, urine	At least 200 candidate biomarkers are found to correlate with disease severity and motor scores	Modulation of different circulating proteins after treatment with nusinersen

PBM, peripheral blood mononuclear cells; CSF, cerebrospinal fluid.

## 2. Circulating biomarkers in SMA

A biomarker is defined as a characteristic that is objectively measured, serving as an indicator of normal biological processes, pathologic processes, or biological responses to a therapeutic intervention (as per the USA FDA definition). Biomarkers can be identified as: susceptibility/risk, diagnostic, monitoring, prognostic, predictive, response and safety biomarker, diagnostic, prognostic, predictive and pharmacodynamic (37, 38).

Circulating biomarkers are molecules (proteins, antibodies, genetic material…) that can be measured in body fluids such as blood, serum, or cerebrospinal fluid (CSF). Reliable circulating biomarkers should be easy to quantify and possibly correlated to the patients’ clinical function. They should also effectively predict response to therapy, as well as support early preclinical diagnosis, prognosis and follow-up in treated and not treated patients of all ages (39). For each of the summarized biomarkers, we will include the general state-of-the-art knowledge and an additional focus on their relevance in assessing patients’ response to treatment.

### 2.1. Genetic based biomarkers

#### 2.1.1. SMN2 copy number

##### 2.1.1.1. Diagnostic and monitoring biomarker

The number of *SMN2* copies in an individual is directly linked to their SMN expression level, meaning that a greater number of *SMN2* copies is connected to a less severe phenotype (6). The *SMN2* copy number, when taken into account alongside factors such as the age at symptom onset and the level of motor skills achieved, functions as an additional criterion for classifying individuals with SMA. Consequently, it can be regarded as a robust indicator of disease severity, applicable to both pediatric and adult patients (40). Despite these aspects, the correlation between *SMN2* copy number and patient’s phenotype is not always accurate. There are individuals with 2 copies of *SMN2* and less severe symptoms and inversely patients with 3–4 copies and classified as phenotypically very severe (41). These discrepancies have therefore pushed many groups to identify other potential modifiers of clinical phenotype (42). Some of them act as protective modifiers (for example coronin-1C-CORO1C, neurocalcin-NCALD or calcineurin-like EF-hand protein-CHP1) (43–45). Plastin-3 (PSL3) (46, 47), a F-actin-bundling protein localized on the X-chromosome involved in neurotransmitter release and vesicle recycling, is considered to be a positive phenotype modifier of particular interest (48).

##### 2.1.1.2. Predictive biomarkers after treatment

*PSL3* overexpression has been shown to improve the clinical phenotype both in animal models of SMA (49) and in human patients (50), with subjects coming from the same family and having the same *SMN2* copy number presenting with variable severity according to PSL3 serum levels. Studies on SMA mice have also demonstrated increased efficacy of treatment by nusinersen when combined with overexpression of human *PSL*3 (51), indicating that *PSL3*, as well as other modifiers, should be taken into consideration in the perspective of a personalized management of each patient. However, further studies are warranted to confirm the role of PSL3 as a predictive biomarker, while studies performed in patients that measured mRNA found a differential effect depending on age and sex, making the interpretation of the results complex (52).

#### 2.1.2. Survival motor neuron (SMN) mRNA transcript levels

Two different transcripts have been proposed as biomarkers of disease severity: SMN2–full length (SMN2-FL) and SMN transcript lacking exon 7 (SMN-D7) (53–55).

#### 2.1.3. SMN2–full length

##### 2.1.3.1. Monitoring biomarker

Results from different studies have shown conflicting results about the reliability of SMN2-FL transcript as a biomarker of disease severity. Although some studies reported that SMN2-FL mRNA levels are able to differentiate between patients with SMA and healthy controls and that FL-mRNA levels in peripheral blood cells inversely correlate with disease severity especially in type 1 patients (56, 57), they were not able to distinguish between different disease phenotypes (54), while some other studies found no correlation between SMN2-FL expression and disease phenotype at all (55, 58, 59).

#### 2.1.4. SMN transcript lacking exon 7

##### 2.1.4.1. Monitoring biomarker

Different studies have attempted to correlate SMN-D7 expression levels and clinical severity, but it was repeatedly reported that they are globally similar to those of healthy controls both in peripheral blood cells and fibroblasts (54, 55, 58).

##### 2.1.4.2. Response biomarker

Idem, in a study involving patients treated with nusinersen, an increase in SMN-FL levels in extracellular vesicles blood was observed after 14 months of treatment (60). In the first-in-human study with risdiplam, it was shown that FL *SMN2* mRNA levels and SMN protein levels increase in whole blood after treatment (56, 57). Studies testing the therapeutic potential of a histone deacetylase inhibitors (valproic acid) on human cell lines, did not show significant changes in SMN-FL levels after treatment (61), while in a corresponding phase 2 trial with valproic acid in children and adolescents SMA patients of all types, SMN2-FL levels were unchanged while SMN-D7 levels were significantly reduced (62). Similar contrasting data were also outlined after clinical trials with phenyl-butyrate (63, 64).

#### 2.1.5. SMN protein levels

SMN protein is ubiquitously expressed and detectable in all cell types, making previous attempts to compare SMN levels in cells in blood and CSF challenging due to tissue-related variations (65).

Some studies (54) show that SMN protein levels are high during the perinatal period in SMA mice and decline rapidly by 3 months, remaining low throughout the mice lifespan (66). Similarly, elevated SMN protein levels in prenatal post-mortem tissues were observed in both SMA patients with 2 *SMN2* copies (type 1 and 2 patients) and healthy individuals, with a confirmed decline after birth in healthy new-borns in different disease-related tissues (spinal cord, brain cortex, diaphragm), suggesting the necessity of SMN protein for prenatal development (67, 68).

##### 2.1.5.1. Prognostic biomarker

While a distinct difference in SMN protein levels is evident between SMA mice and healthy controls, in human patients, there is a notable overlap in *SMN2* expression across different SMA types, indicating that the clinical presentation is more closely linked to the quantity of *SMN2* copies rather than the levels of SMN protein in the blood; however, it’s important to note that the predictive capacity of *SMN2* copy number alone is limited in explaining the variability in clinical phenotypes (69). Similar results have been obtained also by Wadmann et al., who describes lower levels of SMN protein in fibroblasts and PBMC of SMA patients compared to healthy controls, without significant differences among SMA types. In this study, they underlined again the relevant differences of SMN protein levels in different tissues, pointing out that, anyway, SMN protein levels in fibroblasts correlate with *SMN2* copy number and could be a potential biomarker of disease prognosis (58).

##### 2.1.5.2. Monitoring biomarker

Findings from a large multicentre natural history study of infantile SMA (inclusion <6 months of age) also demonstrated a reduction in SMN protein levels in the blood of SMA infants compared to age-matched healthy infants (57). However, a later study indicated that SMN protein levels remain stable over time despite rapid changes in motor function (70). Additionally, another group reported stability in SMN mRNA and protein levels over a year, with no correlation between levels and either muscle function or structural muscle integrity as assessed by functional clinical scales and quantitative MRI (qMRI) of thigh muscles (71). Furthermore, in a cross-sectional study assessing *SMN* gene and protein expression in whole blood, there was no difference in *SMN* expression levels or any other gene expression changes correlating with disease severity between SMA and healthy cohorts (72). Overall, these contrasting results highlight the need for cautious analysis of SMN mRNA and protein levels, considering that systemic levels can be influenced by specific tissue distribution, with peripheral blood levels being globally lower than in motor neurons, age of the patient as well as SMA sub-type.

##### 2.1.5.3. Predictive biomarker

Variable *SMN2* mRNA levels and inconsistent SMN protein levels have been noticed among different tissues, such as the spinal cord, central nervous system (CNS) and others, possibly due to limitations in the detection assay (67). Preclinical studies with morpholino antisense oligonucleotides (ASO) showed that a single injection increased SMN protein levels in both SMA and heterozygote mice, but not to the levels of control animals (73). Nevertheless, the systemic delivery and distribution of ASO resulted in a high degree of variability in SMN protein levels among different tissue types, as well as an age-related change in SMN protein levels in all tissues.

In patients treated with risdiplam, an increase of SMN protein in CSF as well as in peripheral blood has been demonstrated as well (74).

Concerning AAV-mediated gene therapy, it has been described that, despite specific CNS tropism of the AAV9 serotype, the vector is potentially able to reach cells all over the body, with high concentrations being found in motor neurons in both animal models and patients. The percentage of circulating SMN protein produced by the transgene is anyway still difficult to estimate (75). Moreover, even if neurons are considered non-dividing cells, implying that the transgene could be express for a long time after delivery, the SMN protein levels of expression in the long term is still under study.

The differences in SMN levels in peripheral tissues and CNS makes it difficult to consider SMN mRNA and protein as relevant biomarkers of the response to therapy, while results proposing it as a pharmacodynamic and target engagement biomarkers seem to me more consistent and encouraging.

### 2.2. Creatinine and creatine kinase

#### 2.2.1. Monitoring biomarker

Even if CK serum levels are often slightly elevated in all SMA types, they are considered too unspecific to be a reliable diagnostic biomarker of the disease. Nevertheless, taken together with other signs and symptoms of LMN degeneration, high CK serum levels may help orienting the diagnosis of SMA.

#### 2.2.2. Prognostic and predictive biomarker

Some of the first potential prognostic circulating biomarkers proposed as useful in SMA were serum creatinine and CK. Serum creatinine levels are known to be directly correlated to lean muscle mass in healthy individuals. Moreover, serum creatinine is currently under investigation as a circulating biomarker in other MNDs, such as amyotrophic lateral sclerosis (ALS) and spinal-bulbar muscular atrophy (SBMA), where it shows an inverse correlation with the severity of denervation (76–78). In rapidly progressive forms of SMA, creatinine serum levels have been found to be inversely associated with disease severity after adjusting for age and lean mass (34), with higher levels observed in type 3 patients then in type 1. The amount of creatinine declines over time regardless of the SMA type and seems to be a slightly more reactive biomarker than neurophysiological parameters in pre-symptomatic children (79). On the other side, in chronic forms of the disease concerning mostly adolescents and adult patients, creatinine serum levels are considered to be higher than in patients with rapidly progressive forms (79).

CK is an enzyme that catalyzes the reversible transfer of phosphate to creatine, generating phosphocreatine, which serves as a mobilizable energy reserve in skeletal muscle. Increased CK levels have been described especially in milder chronic forms of SMA, including adolescent and adult patients, probably in relation to greater muscle mass (80).

In patients treated with nusinersen, a decrease in CK and a parallel increase in serum creatinine after several months of treatment were observed both in children and adult patients (80, 81). Additionally, serum creatinine and CK levels were higher in responders than in non-responders (82). These findings suggest that serum creatinine and CK levels can be considered easy measurable response biomarkers for assessing response to therapy in SMA.

As anti-myostatin treatments are under study in clinical trials, aiming at directly targeting muscle trophism and strength, the role of creatinine and CK levels, as well as that of other markers of muscle activity, will be of primary interest to provide biological evidence of response to therapy (25).

## 3. Neurofilament heavy and light chain

Neurofilaments (Nfs) are a group of cytoskeletal proteins belonging to the type IV intermediate filaments family. They consist of three subunits: neurofilament light chain (NfL, 68 kDa), medium chain (NfM, 150 kDa) and heavy chain (NfH, 200 kDa) (83). Nfs are uniquely expressed in neurons, and play a crucial role for axon radial growth and axonal regeneration (84). They are abundant in large, myelinated axons, where they are highly organized into parallel arrays (85). Nfs undergo post-transcriptional regulation processes such as phosphorylation (referred to as p-Nf), which enhances their resistance to degradation (86). However, once they are released following neuronal loss or axonal damage, they can be detected into the CSF, peripheral blood and interstitial fluid (87–89). In light of this background, Nfs have emerged as promising biomarkers for several neurological and neurodegenerative diseases including other motor neuron diseases such as ALS. In ALS, Nfs were demonstrated to be elevated at all disease stages as well as in preclinical carriers of specific gene mutations, suggesting that they could be used as an effective biomarker of disease severity and progression but also of response to future therapies (90–94).

As per existing models of Nfs production and circulation, CSF and blood levels of these proteins are elevated in young, healthy children (95), with a tendency to increase during the early phases of CNS development. Subsequently, both CSF and plasma levels demonstrate a stabilization, followed by a decline in late childhood and adolescence. However, in the context of healthy aging, a slight increase is observed once again. Specifically, within the CSF, the upper reference value for NfL levels shows a 2.5-fold increase between the ages of 20 and 50, and then doubles by the age of 70 (96). This trend could be justified by various factors, including a slowdown in Nfs metabolism, ongoing neural loss due to the aging process, subjects’ body mass index (BMI) and renal function (97).

### 3.1. Monitoring and prognostic biomarker

In children with SMA type 1, plasma levels pNf-H and Nf-L exhibit a substantial increase during the initial months of life when compared to age-matched controls, indicating active and rapid motor neuron degeneration and death at early stages of the disease (83, 98, 99). Additionally, serum Nfs levels are inversely associated with *SMN2* copies number, and directly correlate with earlier age of diagnosis and symptom onset and lower baseline motor function (34). Over time, pNf-H levels decline in untreated SMA children, but persistently remain higher compared to healthy controls of the same age, probably due to the active axonal degeneration (99). Recent data have revealed notable fluctuations in pNf-H levels among both SMA patients and mouse models, highlighting the necessity for a comprehensive interpretation of this biomarker’s measurements (100). However, in the context of adolescent and adult patients, the significance of measuring Nfs as informative biomarkers is less clear. Data from published studies indicate that adolescent and adult patients with type 2 and 3 SMA globally show no significant CSF and serum/plasma increase in Nfs compared to age-matched controls, (101–104). A recent paper also suggested that, in a similar cohort of adult SMA type 2 and 3 patients, pNf-H levels in the serum were reduced compared to healthy controls, possibly reflecting motor neuron pool exhaustion as a result of long lasting chronic degeneration (100).

### 3.2. Response biomarker and surrogate end-point

In pre-symptomatic and symptomatic children, pNf-H has been proposed as a reliable biomarker of response to treatment from the first clinical trial with nusinersen [ENDEAR (22)], which demonstrated a rapid decrease in pNf-H circulating levels within 2 months of intrathecal injections. This decline was also noticeable in the control group, albeit at a slower pace and with less pronounced effect, often followed by stabilization. Intriguingly, treated patients exhibited consistently higher pNf-H levels in both serum and CSF compared to age-matched healthy individuals across all time points despite treatment. Two additional studies of non-ambulatory SMA type 1 infants with 2 *SMN2* copies receiving nusinersen therapy further confirmed the rapid decline in Nfs in the CSF, with additional evidence of direct correlation of Nfs levels with clinical presentation, supporting the utility of Nfs as predictive, response and pharmacodynamic biomarkers (105–107).

Among adult patients, a general consensus exists on the fact that these levels do not change after treatment with nusinersen (102, 103) and that a clear correlation with motor improvement cannot be confirmed (108, 109). Interestingly, higher serum Nf-L levels appear to be linked to poorer motor performance, though changes in motor function generally do not correspond with shifts in serum Nf-L. A further confounding factor in adolescent and adult type 2 and 3 patients is that the disease presents slow progression and high clinical heterogeneity. Moreover, treatment availability with nusinersen only started in 2016, implying that adult patients were treated after an extremely variable disease duration, further complicating the possibility to perform reliable statistical analysis of Nfs levels correlation with disease stage and progression (110). However, it is known that younger type 3 patients have a more evident and significant decline of pNf-H and Nf-L levels in the CSF after 3 nusinsersen loading doses (111).

No data are available about Nfs change in patients treated with Risdiplam, in both pediatric and adult patients. Notably, a recent study showed an unexpected increase of Nfs in 7 pre-symptomatic patients SMA infants treated with intravenous gene therapy between 20 and 190 days of age regardless of *SMN2* copy number (83). At the same time, infants that were pretreated with nusinersen as a co-therapy within a short interval before their onasemnogene abeparvovec infusion did not show an increase in Nfs levels. One child carrying 2 *SMN2* copies, presented incomplete benefit from gene therapy and developed mild symptoms of motor neuron degeneration over time. This individual demonstrated chronically elevated Nfs levels that failed to normalize during the subsequent 18 months after therapy. While the interpretation of these findings should be cautious, one hypothesis could be that nusinersen could have a neuroprotective effect, paving the way for new combination therapy strategies to come.

## 4. Micro-RNA

MicroRNAs are a class of small (~22 nt) non-protein-coding RNA molecules that can regulate gene expression at a post-transcription level (112, 113). More specifically, miRNA bind to determined sequences in the 30-UTR of target genes, inducing subsequent translational repression and/or decay of target mRNAs (114). Endogenous non-coding RNAs also regulate gene expression via formation of a ribonucleoprotein (RNP) complex with Argonaute (AGO) proteins and complementary base pairing with their target mRNAs (115).

### 4.1. Pathophysiology and monitoring biomarker

In the latest years of research on microRNAs, their key role in the regulation of muscle and CNS development and regeneration has emerged, placing them within the possible non-invasive biomarkers in neurological and neuromuscular diseases such as SMA (35, 116, 117). Moreover, it has been shown that SMN protein has a critical role in miRNA biogenesis and processing, confirming the relevance of miRNAs in SMA pathogenesis. Their dysregulation is not only a consequence of the disease, but it can contribute to exacerbate its pathological features (118). Given their role in the pathogenesis of SMA, it is possible that miRNA expression changes after disease-modifying treatments, suggesting that they could be a biomarker of response to therapy as well (119). miRNAs are relatively stable in accessible biofluids, such as blood and CSF, and can be detected with accessible and feasible laboratory methods.

Differential expression of different miRNAs (miR-9, miR-206, miR-34, miR-132, miR-225-5p, miR-431, miR-375…) have been reported in SC, skeletal muscle, and serum from SMA and wild-type mice as well as in serum samples from SMA and control patients (114, 119). Mice lacking the miRNA-processing enzyme Dicer selectively in motor neurons display hallmarks of SMA (120). Also, SMN protein has been shown to alter miRNA expression and distribution in neurons. For example, SMN protein down-regulates the expression of miR-9a and, interestingly, miR-9a levels in fibroblasts have shown a positive correlation with SMA severity (121). miR-9 alteration leads to reduced down-regulation of heavy Nfs in motor neurons (114, 121). In fact, neurofilament accumulation has been supported by several studies as one of the more likely causes of selective motor neuron degeneration. The neurons that are most prominently affected by the accumulation of neurofilaments are the largest neurons with the longest axons, such as motor neurons the most vulnerable cells in motor neuron disorders, including SMA (122).

miR-183 is known to be increased in neurites of SMN-deficient neurons. Inhibition of miR-183 expression in the spinal cord of an SMA mouse extends survival and improves motor function of Smn-mutant mice (123).

miR-431, involved in motor neuron neurite length, also plays a role in the SMA motor neuron phenotype. Its expression is namely highly increased in spinal motor neurons and a number of its putative mRNA targets are significantly down-regulated in motor neurons after SMN loss (117).

Another miRNA involved in SMA motor neuron phenotype is miR-375. Besides its role in neurogenesis, miR-375 protects neurons from apoptosis in response to DNA damage. Motor neurons from a SMA patient have shown reduced levels of miR-375, elevated p53 protein levels, and higher susceptibility to DNA damage induced apoptosis (124).

miRNAs could be useful also to better understand and track muscle involvement in SMA. Some miRNAs strictly related to muscle function (including miR-1, miR-133a, miR-206), which are up-regulated by the action of MYOD protein in myoblasts, are reduced in SMA myoblasts since MYOD1 is reduced as well as a result of *SMN1* mutations, resulting in impaired metabolism and myotubes formation (116). These results suggest a role of SMN in regulation of myogenesis (125) and propose that specific miRNAs could be effective biomarkers of delayed process in SMA mouse models and patients.

### 4.2. Predictive and response biomarker

In infants treated with nusinersen, miRNA dosing has been performed on CSF and blood samples, showing how some of them were longitudinally reduced before and after treatment (for example miR-378, known to regulate the balance between myocyte autophagy and apoptosis) (126). miR-378a-3p has been found elevated after treatment especially in patients with more important motor improvement. Similarly, higher baseline levels of miR-142-5p and miR-355-5p seem to be associated with better reaction to nusinersen treatment. On the other side, miR-23a, which has been hypothesized to have a neuroprotective function, has been found in higher concentrations in blood from patients presenting the best response to nusinsersen treatment both at baseline and longitudinally (127).

The myomiRNAs miR133a, 133b, 206 and 1 were demonstrated to be downregulated upon nusinersen treatment in the serum of SMA pediatric patients, with the levls of miR-133a being correlated with motor function improvement. It remains to be established if this miRNA is modulated in adult patients’ treatment (128). In other studies, baseline levels of miR206 negatively correlated with patients’ response (122, 129) suggesting the importance of studying the myomiRNAs before and after therapy. Studies testing a single dose therapeutic morpholino antisense oligomer treatment in SMA mice showed that miR-132 levels (which are known to be pathologically elevated in SMA mice) in the SC, muscle and serum, reversed to the normal levels after treatment, suggesting that miR-132 could be one of the most responsive miRNAs to systemic ASO treatment in the severe mouse model (129).

A very recent study additionally highlighted the importance of miR34 family (miR34a/b/c) in MN functional regulation and has positively correlated this miRNA to the type 1 SMA patients’ response to nusinersen treatment (130). The authors have further demonstrated a therapeutic effect of miR34a administration in pre-symptomatic SMA mice substantiating the importance of this miRNA in the pathophysiology and treatment of SMA.

Altogether, miRNA seem to be encouraging possible biomarkers both of disease severity and response to treatment in SMA patients and animal models. Moreover, they are relatively easy to measure in CSF and blood, permitting iterative sampling in treated patients. However, information about role and function of different miRNA is still under study, and their concentration could change in different tissues as well as with development and aging. Cautions should then be applied in the interpretation of results describing the role of single miRNA especially in treated patients since repetition studies are not available yet and actual results are highly heterogenous. Probably, a combination of different miRNA could hold an interesting value in prediction of disease evolution and response to therapeutic interventions.

## 5. Omics and other circulating proteins

Over the years, several studies focusing on identifying possible circulating proteins that could represent a reliable biomarker in SMA have been performed. The Biomarkers for SMA (BforSMA) study is an example of this effort. It was a cross-sectional omics study that evaluated blood and urine protein analytes in children (age 2–12 years) with genetically confirmed SMA and age-matched healthy controls (60). A resulting 200 candidate biomarkers were found to correlate with motor scores, and the most significant markers across all outcome measures were plasma protein analytes. The novelty of this study consisted in the use of several unbiased methods (metabolomics, proteomics, and transcriptomics) for the search of biomarkers in a large well-defined SMA patient cohort and age-matched healthy controls. Despite the identification of several putative biomarkers, further validation is needed to confirm these findings.

### 5.1. Predictive biomarker

Some recent studies have analyzed the role of proteomics as a biomarker in SMA patients treated with nusinersen (131, 132), suggesting that the treatment could modulate the protein profile of the CSF. Further studies are warranted to better define which proteins are the most reactive to treatment both in blood and CSF.

Results from studies in non-treated and treated patients are resumed in Table 2.

**Table 2:** Overview of multi-omics approaches used to date to characterize disease progression in non-treated and treated SMA patients.

References	Omics	Sample’s source	Highlights	Treatment
Tosi et al. (133)	Transcriptomic	iPSCs-derived motorneurons from SMA patients and healthy controls	NRXN2 protein downregulation was identified	NA
Hijikata et al. (77)	Metabolomic, transcriptomic, proteomic	Plasma and urine from 108 SMA type 1, 2 and 3 patients (2–12 years of age)	97 proteins and 59 metabolites in the plasma together with 44 metabolites in the urine correlated with functional score	NA
Jones et al. (134)	Transciptomic	Spinal motoneurons isolated from human CNS sections from SMA patients	Synaptogamin13 was identified as a putative neuroprotective protein in MND	NA
Roberto et al. (151)	Proteomic	Extracellular vesicles released from fibroblasts	116 statistically significant protein alterations compared to control cells	NA
Varderidou-Minasian et al. (152)	Proteomic	iPSC from healthy individuals and SMA patients	Profile of several SMN-binding partners	NA
Brown et al. (153)	Proteomic	Fibroblasts from type 1, 2, and 3 SMA patients	PYGB (SMA I), RAB3B (SMA II), and IMP1 and STAT1 could correlated with disease severity	NA
Chen et al. (130)	Proteomic	CSF 10 Nusinersen-treated adults SMA type 2 and 3 patients followed-up over 10 months	No correlation between protein profiling and functional score evolution	Nusinersen
Schorling et al. (154)	Proteomic	CSF if 31 type 1 Nusinersen-treated patients after 2 months from beginning of the treatment	Downregulation of cathepsin D after treatment	Nusinsersen
Magen et al. (129)	Proteomic	CSF of 10 type 1SMA patients (2–28 months) followed-up overt 6 months	Marked up-regulation of apolipoprotein A1 and apolipoprotein E transthyretin	Nusinsersen

CSF, cerebrospinal fluid; MND, motor neuron diseases; iPSC, induced pluripotent stem cells; CNS, central nervous system.

## 6. Discussion

SMA is a complex disease characterized by heterogeneous clinical presentation. The recent emergence of several effective drugs and ongoing research into novel therapies highlights the pressing need for reliable biomarkers to guide patient-specific therapeutic strategies. In this context, identifying biomarkers that are accurate but also easily implementable in clinical trials and practice becomes imperative. Furthermore, these biomarkers should be closely associated with therapy response to facilitate informed decisions regarding treatment initiation, cessation, or modification (35, 36). Clinical and functional measurements, as well as imaging and neurophysiology, surely play a relevant role in patients’ classification and in monitoring disease progression (29, 32, 135–139). Nevertheless, circulating biomarkers, including circulating proteins, Nfs and other small molecules such as miRNA, might allow better categorization of patients of all disease sub-types and present great potential to detect significant modifications after treatment, namely in adolescent and adult patients with a relatively stabilized and slowly progressive motor deficit. From a practical point of view, circulating biomarkers are generally easy to access and less operator-dependent (39), allowing repeated measurement over time in treated and non-treated patients.

Among the various putative circulating biomarkers under investigation, Nfs stand out as promising candidates, particularly in children with type 1 and 2 SMA (83, 98, 99). These biomarkers have exhibited favorable responsiveness to treatments such as nusinersen and gene therapy. Their utility extends to monitoring both therapy response and disease progression over time, as they can be assessed in CSF and blood samples. This dual applicability makes neurofilaments valuable tools for tracking disease dynamics and therapeutic efficacy in pediatric SMA patients (83). However, it is important to note that the utility of neurofilaments in adult SMA patients appears limited. In the adult population, alterations in neurofilament levels are only marginal, and the changes following nusinersen therapy are less pronounced and harder to interpret (102, 104, 111). This discrepancy between pediatric and adult populations emphasizes the need for age-specific biomarker considerations when making therapeutic decisions in SMA.

Traditionally, *SMN2* copy number has been regarded as the principal genetic modifier in SMA, significantly influencing disease severity (10). Nevertheless, recent research has shed light on the existence of additional positive modifiers, such as PSL3 (46), which are currently under investigation. These emerging modifiers hold the potential to play a role in shaping the phenotype’s severity, necessitating their inclusion in decision-making processes for therapy choices as well as in helping setting patients and families’ expectations. As our understanding of SMA genetics evolves, these modifiers may offer new insights into the disease’s pathogenesis and treatment response.

In contrast to neurofilaments and genetic modifiers, SMN protein and SMN-mRNA appear to be promising predictive and pharmacodynamic biomarkers for systemic treatments in both pediatric and adult SMA patients (55). However, their applicability to intrathecal treatments is limited. This distinction underscores the importance of tailoring biomarker selection to the specific treatment modality employed in SMA patients, further highlighting the complexity of individualized therapy decisions.

In conclusion, the pursuit of effective SMA therapies underscores the critical role of biomarkers in guiding treatment decisions. Neurofilaments emerge as valuable biomarkers in pediatric populations, reflecting both therapy response and disease progression. In contrast, *SMN2* copy number and other emerging modifiers like PSL3 are essential considerations for understanding disease severity and therapeutic implications. The distinctions in biomarker utility between pediatric and adult populations, as well as across treatment modalities, emphasize the need for a nuanced and patient-specific approach to SMA management. As research in this field continues to advance, the integration of multifaceted biomarker data will be essential for optimizing therapeutic outcomes in individuals with SMA.

The development of informatic and especially artificial intelligence (AI) tools might also be useful in the categorization of more relevant circulating biomarkers as well as in the development of meaningful biomarkers combinations that could, in the end, be more robust than single biomarkers. Such approach has already been demonstrated to be effective in other pathologies such as cancer or infectious disease (140–142). In this perspective, the development of multimodal tools composed both by clinical and functional and by molecular and circulating biomarkers could improve our ability to predict prognosis and reaction to treatment (36, 137). Composite scores should moreover be adapted to patients’ age, phenotype and to different treatment approaches and have the potential to substantially improve the clinical knowledge and the patients’ care.

Recent approval of three disease-modifying therapies for SMA (143–149) opened the door to great improvements in patient survival and development of new clinical phenotypes. Moreover, it is now well known that treatment must be initiated as early as possible to potentially improve the patient’s response to it. However, phenotypic heterogeneity and the wide spectrum of treatment response still make the decision process and the choice of the most adapted treatment for each patient challenging, adding even more importance to the study and development of new robust biomarkers to guide patient-targeted intervention.

On one side, biomarkers are useful in stratifying patients before therapy administration, helping in the choice between different therapeutic approaches, while, on the other, they are necessary to monitor disease progression under treatment. This is of primary importance in clinical trials to demonstrate the efficacy and safety of new molecules, but also in clinical practice, especially as combination therapies (for example, gene therapy followed by nusinersen or risdiplam), or switch from one treatment to another are proposed to patients with only partial response to a first therapeutic approach (23, 133, 150). With new and different treatments on their ways (for example anti-myostatin drugs), the ability to stratify patients will further help in the decision of when and how to combine therapies.

In this context, individualized treatments choices will gain more importance in the aim of developing a personalized approach specific to each patient. It is in fact nowadays known that response to treatment, but also natural history evolution, can largely differ from one patient to another even in the same family (134) further underlying the need for more precise and specific biomarkers to better characterize patients’ phenotype as well as to predict prognosis with and without treatment.

## Author contributions

QG, MG, and PS: Conceptualization, Writing—original draft preparation, Writing—review and editing. QG: Revision and funding acquisition. All authors have read and agreed to the published version of the manuscript. All authors contributed to the article and approved the submitted version.

## Figures and Tables

**Table:** 

AAV	Adeno-associated virus
AGO	Argonaute protein
ALS	Amyotrophic lateral sclerosis
ASO	Antisense oligonucleotide
BMI	Body mass index
CHP1	Calcineurin-like EF-hand protein
CK	Creatine-kinase
CSF	Cerebrospinal fluid
CNS	Central nervous system
CORO1C	Coronin-1C
DNA	Deoxyribonucleic acid
EMA	European Medicines Agency
FDA	Food and Drug Administration
FL	Full length
iPSC	Induced pluripotent stem cell
LMN	Lower motor neuron
MND	Motor neuron disease
MRI	Magnetic resonance imaging
mRNA	Messenger-RNA
miRNA	Micro-RNA
NCALD	Neurocalcin
Nfs	Neurofilaments
NfH	Neurofilament heavy chain
NfM	Neurofilament medium chain
NfL	Neurofilament light chain
PBMC	Peripheral blood mononuclear cells
p-NF	Phosphorylated neurofilament
PSL3	Plastin-3
q-MRI	Quantitative MRI
RNA	Ribonucleic acid
SBMA	Spinal-bulbar muscular atrophy
SC	Spinal cord
snRNP	Small nuclear ribonucleoprotein
SMA	Spinal muscular atrophy
SMN	Survival motor neuron
USA	United Stated of America
